# Effectiveness of Intravenous Iron Sucrose Therapy in Routine Antenatal Care for the Treatment of Moderate to Severe Anemia Among Pregnant Women Attending a Secondary Care Hospital in North India: A Retrospective Analysis

**DOI:** 10.7759/cureus.23603

**Published:** 2022-03-29

**Authors:** Ravneet Kaur, Shashi Kant, Muthathal Subramanian, Bhushan D Kamble, Sumit Malhotra, Partha Haldar

**Affiliations:** 1 Centre for Community Medicine, All India Institute of Medical Sciences, New Delhi, IND; 2 Community and Family Medicine, All India Institute of Medical Sciences, Raipur, IND; 3 Community and Family Medicine, All India Institute of Medical Sciences, Bibinagar, IND

**Keywords:** pregnant women, parenteral iron therapy, hemoglobin, intravenous iron sucrose (ivis), anemia

## Abstract

Introduction: Anemia during pregnancy is an important public health problem and is associated with a number of maternal and fetal complications. Intravenous iron sucrose (IVIS) has been reported to be safe and efficacious in raising the hemoglobin (Hb) level among pregnant women. However, most of the studies were conducted in controlled research settings, and there is a paucity of data on the effectiveness when IVIS is given as routine therapy in public health facilities. The objective of this study was to estimate the change in mean Hb level four weeks after the last dose of IVIS infusion in pregnant women with moderate to severe anemia in a public healthcare setting.

Methods: Records of pregnant women having moderate anemia (Hb level: 7.0-9.9 gm/dL), who received IVIS in calculated dose during routine antenatal care between 1 January 2018 and 31 July 2018, were reviewed. Data were analyzed using STATA version 13 software (StataCorp LLC, College Station, TX). Hb levels before the start of the therapy (baseline) and four weeks after the last infusion (endline) were compared. A value of p < 0.05 was considered statistically significant.

Results: The mean (±SD) Hb level increased from 8.5 (±0.88) gm/dL at baseline to 10.3 (±1.24) gm/dL four weeks after the last dose of IVIS infusion. The mean (±SD) increase in Hb level was 1.7 (±1.29) gm/dL (95% CI: 1.57, 1.87). The change from moderate and severe anemia to normal Hb levels was observed in 28.4% and 28.6% of women, respectively.

Conclusion: IVIS therapy is effective in improving Hb levels when given as routine therapy in a secondary level public healthcare facility.

## Introduction

Anemia during pregnancy is associated with complications such as post-partum hemorrhage, low birth weight, premature births, stillbirths, and maternal deaths [1]. The World Health Organization (WHO) estimates that nearly 40% of pregnant women and one-third of all women of reproductive age worldwide are anemic [2]. In India, the National Family Health Survey, 2019-2021 (NFHS-5) reported that 52.2% of pregnant women in India were anemic, the prevalence being higher in rural areas (54.3%) than in urban areas (45.7%). The study was conducted in the Faridabad district of Haryana state, where the reported prevalence of anemia among pregnant women was 55% [3]. Iron deficiency is the most common cause of anemia and is estimated to contribute to approximately 50% of all cases of anemia among non-pregnant and pregnant women worldwide [4]. Oral iron supplementation (iron-folic acid tablets) is the therapy of choice for prophylaxis and treatment of mild and moderate iron deficiency anemia in pregnancy [5]. However, oral iron therapy requires a prolonged duration of treatment, which is often beset with poor compliance [6]. Therefore, parenteral iron therapy is an alternative treatment modality for pregnant women with moderate anemia [7]. Intravenous iron sucrose (IVIS) has been reported to be safe and efficacious in rapidly raising the hemoglobin (Hb) level among pregnant women [8-10]. However, most of these studies were conducted in tertiary care centers under controlled research settings. There was a paucity of data on the effectiveness of IVIS therapy seen under field conditions, i.e., when IVIS is given as routine therapy in public health facilities. Secondary care hospitals are the most likely setting where IVIS would be administered. Hence, we conducted this study to estimate the effectiveness of IVIS when given as routine therapy to pregnant women with moderate to severe anemia who attended an antenatal clinic at a secondary care hospital.

## Materials and methods

Study design and setting

We did a retrospective analysis of the clinical data recorded as part of the hospital services. The study site was Sub-District Hospital (SDH), Ballabgarh, which is a secondary care hospital located in the Faridabad district of Haryana, India. The SDH is a 50-bed hospital. An outpatient antenatal care (ANC) clinic was held thrice a week. On average, approximately 300 deliveries took place every month in the hospital.

Pregnant women (>12 weeks of gestation) who attended the ANC clinic and were moderately anemic (Hb level: 7.0-9.9 gm/dL) were offered IVIS infusion during the daycare admission in the ward. Women who were known to be hypersensitive to iron formulations were transfused blood or blood products in the preceding three months and women who suffered from any chronic disease or hematological disorders were not provided IVIS. Pregnant women with Hb levels <7.0 gm/dL were referred to the district hospital for blood transfusion. Those pregnant women with Hb level <7.0-5.0 gm/dL, who did not agree to blood transfusion, were then offered IVIS in their best interest. The total iron requirement was calculated using the Ganzoni formula [11]. The calculated dose was rounded off to the nearest hundred. IVIS was infused as 300 mg of elemental iron diluted in 300 mL of normal saline over a period of one hour per sitting on alternate days to a maximum of 600 mg per week. Hb level was measured by a trained nurse, using a digital hemoglobinometer (HemoCue 201+, HemoCue AB‑Hb Photometer, Angelholm, Sweden) [12]. HemoCue 201+ has reported a sensitivity of 93% and specificity of 73% compared to auto-analyzer [13]. For quality assurance, controls were run after every 30 samples were tested.

Hb level prior to the start of IVIS therapy was considered as baseline level and four weeks after the last IVIS infusion as endline Hb level. Data collection and collation were done by the study investigators (MS and BK), while the study process was facilitated and supervised by RK, SK, PH, and SM.

Eligibility criteria

All pregnant women who had received IVIS during the period from 1 January 2018 to 31 July 2018 and for whom both baseline and endline Hb levels were available were included in the study.

Sample size

As reported in a previous study [10], the minimum mean difference in Hb level between baseline and endline was assumed to be 1 gm/dL. With a standard deviation (SD) of 1.3 and power of 95%, the minimum calculated sample size was 90 women. Since IVIS therapy involves multiple visits and the study was conducted in a public healthcare setting, we adjusted for a high rate of attrition (35%), and the final sample size was calculated to be 138. We analyzed the data of 282 women. Hence, the study was adequately powered.

Sources of data

The IVIS register available in the ward contained the details of pregnant women who had received IVIS. Hb measurements were available in the ANC outpatient department register. Socio-demographic and obstetric history were obtained from ANC cards available in the ANC clinic. The category of anemia was as per the Government of India guidelines [7].

Statistical analysis

Data were entered into Microsoft Excel (Microsoft Corporation, Redmond, WA) and analyzed using STATA version 13 (StataCorp LLC, College Station, TX). Descriptive analysis of the change in Hb level from baseline to four weeks after the last dose of IVIS is presented as mean (±SD) along with a 95% confidence interval (CI). Paired t-test was applied and a value of p < 0.05 was considered statistically significant.

Ethical issues

The study protocol was approved by the Institutional Ethics Committee, All India Institute of Medical Sciences, New Delhi vide letter number IEC-646/07.12.2018. Information collected during the study was kept confidential.

## Results

A total of 282 pregnant women who were either moderately or severely anemic were included in the study. The mean (±SD) age was 24.3 (±3.4) years, and the range was 19-40 years. A small proportion (10%) of pregnant women had not received any formal education. Most (94.3%) of the pregnant women were homemakers. Almost one-third (28.4%) of the pregnant women were primigravida. The mean (±SD) period of gestation (POG) at the time of enrolment in the study was 22.1 (±5.6) weeks. The majority (85.1%) of the pregnant women were in the second trimester of pregnancy (Table 1).

**Table 1:** Distribution of pregnant women by selected socio-demographic and obstetric variables (N = 282). * Eighty women were primigravida; data were missing for 24 women.

Variable	Category	Number	Percentage
Age group (completed years)	20 or below	31	11
	21-25	160	56.7
	26-30	80	28.4
	31-35	9	3.2
	36-40	2	0.7
Years of education	No formal education	28	10.0
	5 or less	30	10.6
	8	63	22.3
	10	48	17.0
	12	53	18.8
	More than 12	60	21.3
Occupation	Homemaker	266	94.3
	Working women	16	5.7
Gravidity	1	80	28.4
	2	93	33
	3	79	28.0
	4 or more	30	10.6
Number of abortions	0	214	75.9
	1	51	18.1
	2 or more	17	6.0
Average spacing (between two children)* in years (n = 178)	1-2	54	30.3
	2.5-5	100	56.2
	5-7.5	13	7.3
	8-10	11	6.2
Period of gestation in weeks	12-28	240	85.1
	29-32	34	12.1
	32 and above	8	2.8

The mean (±SD) iron requirement was 996 (171) mg. The number of prescribed doses ranged from one to five, with a mean (SD) of 3.32 (0.57). The majority of the pregnant women (91.5%) completed all the prescribed doses. A total of 24 (8.5%) pregnant women did not complete the prescribed doses. Out of these, 15 (62.5%) pregnant women missed one dose, four missed two doses, and five missed three doses.

The mean (±SD) Hb level at baseline was 8.5 (±0.88) gm/dL. The majority (95.0%) of the pregnant women had moderate anemia. There were 14 (5.0 %) pregnant women who had severe anemia. The baseline mean (±SD) Hb levels in women who had moderate and severe anemia were 8.7 (±0.75) and 6.4 (±0.45) gm/dL, respectively.

The overall mean (±SD) Hb level increased from 8.5 (±0.88) gm/dL at baseline to 10.3 (±1.24) gm/dL four weeks after the last dose of IVIS infusion. The mean (±SD) increase in Hb level was 1.7 (±1.29) gm/dL (95% CI: 1.57, 1.87). This difference was statistically significant (p < 0.001). The mean increase in Hb level among pregnant women with severe anemia at baseline was twice as high (3.5 gm/dL) in comparison to the women who were moderately anemic (1.6 gm/dL) (Table 2).

**Table 2:** Distribution of change in hemoglobin (Hb) level (in gm/dL) by category of anemia at the baseline assessment (N = 282). * Hemoglobin = 7.0-9.9 gm/dL; # hemoglobin < 7.0 gm/dL.

Category of anemia at the baseline assessment (n)	Baseline Hb, mean (SD)	Baseline Hb, 95% CI	Endline Hb, mean (SD)	Endline Hb, 95% CI	Change in Hb, mean (SD)	Change in Hb, 95% CI	P-value
* Moderate (n = 268)	8.7 (0.75)	8.57-8.75	10.3 (1.25)	10.14-10.45	1.6 (1.22)	1.48-1.77	<0.001
# Severe (n = 14)	6.4 (0.45)	6.14-6.66	9.9 (1.20)	9.24-10.63	3.5 (1.16)	2.86-4.20	0.001*

Out of 268 pregnant women with moderate anemia, 76 (28.4%) attained normal Hb levels, 95 (35.4%) improved to the mild category of anemia, while 97 (36.2%) remained in the moderate anemia category. None of the women regressed to the severe anemia category. Among 14 women who had severe anemia, four (28.6%) became non-anemic, another four (28.6%) improved to mild anemia, while six (42.8) moved to moderate anemia category at the time of endline Hb measurement.

The change from moderate anemia to normal Hb level, from severe to normal Hb level, and the overall change from anemia to the non-anemic category was observed in 28.4%, 28.6%, and 28.4% of the pregnant women, respectively (Table 3).

**Table 3:** Distribution of change in the category of anemia at the time of endline hemoglobin (Hb) level measurement.

Category of anemia at the baseline assessment	Category of anemia at the endline assessment			
	No anemia (Hb ≥ 11 gm/dL), n (%)	Mild (Hb = 10.0-10.9 gm/dL), n (%)	Moderate (Hb = 7.0-9.9 gm/dL), n (%)	Severe (Hb < 7.0 gm/dL), n (%)
Moderate (n = 268)	76 (28.4)	95 (35.4)	97 (36.2)	NIL
Severe (n = 14)	4 (28.6)	4 (28.6)	6 (42.8)	NIL
Total (N = 282)	80 (28.4)	99 (35.1)	103 (36.5)	NIL

## Discussion

The reported increase in Hb level following administration of IVIS ranges between 1.6 and 3.6 gm/dL [8-10]. The wide range is probably reflective of the differences in patient characteristics, dose and schedule of administration of IVIS, timing, and method used to measure the Hb level. In our study, 28.4% of pregnant women attained normal Hb levels four weeks after the last dose of IVIS administration. Our finding was in agreement with a study by Thakor et al. [14], where the corresponding figure was 24%. However, Kriplani et al. [15] reported that a normal Hb level was achieved by 67% of the participants. However, the mean increase in Hb level was similar to the present study. It, therefore, appears that the observed difference could be due to the differences in participants’ characteristics, particularly the baseline Hb level. The efficacy reported by Kriplani et al. was in controlled research conditions in a tertiary care hospital. In contrast, our study was under routine ANC service at a secondary care hospital. Hence, the observed difference could be due to a different study setting.

We found that the overall mean (±SD) Hb level increased from 8.5 (±0.88) gm/dL at baseline to 10.3 (±1.24) gm/dL at the end of four weeks. The overall mean difference was 1.7 gm/dL (95% CI: 1.57, 1.87). This difference was statistically significant (p < 0.001). The mean increase in Hb level in our study was similar to the study by Haldar et al. [10] that was conducted in a rural area of Ballabgarh, Faridabad. Various studies on the effectiveness of IVIS report mean gain in Hb levels ranging from 1.6 to 3.6 gm/dL in different parts of India [16-18].

We had included 14 pregnant women with severe anemia (at baseline). None of these pregnant women remained severely anemic at the time of endline assessment of Hb level. The mean rise in Hb level for women who were severely anemic at the baseline was twice as high (3.5 gm/dL) in comparison to the women who were moderately anemic (1.6 gm/dL). This meant those with more severe disease conditions benefitted most. However, Haldar et al. had reported that the mean increase in Hb level in the severely anemic group was 2.5 gm/dL. This difference may be due to the total amount of elemental iron infused into a pregnant woman. We used the Ganzoni formula to calculate the total iron requirement. Haldar et al. had infused a fixed amount of 400 mg of elemental iron divided into four doses of 100 mg each. The mean elemental iron administered in our study was 2.5 times higher (996 mg) compared to the study by Haldar et al. [10].

The quantum of increase in Hb level among pregnant women with severe anemia was clinically meaningful. All of them moved to a milder category of anemia. A little more than a quarter became non-anemic. All this was achieved within a short span of time, i.e., four weeks after the last dose of IVIS infusion. Most of the serious maternal and fetal consequences of anemia are concentrated among pregnant women with severe anemia. Hence, we feel that in situations where blood transfusion is unavailable or refused, IVIS infusion for pregnant women with severe anemia would be a viable alternative.

Limitations

Since it was a retrospective record review, there were some missing data. We could not comment on the replenishment of body iron reserve due to the non-availability of data regarding serum ferritin levels. Due to the research design, we did not have a comparator arm. Therefore, direct evidence of a causal relationship between IVIS administration and mean increase in Hb level could not be established.

## Conclusions

Infusion of IVIS to moderately anemic pregnant women in a routine ANC clinic increased the mean Hb level four weeks after the last infusion. The magnitude of increase in mean Hb level was twice as much in pregnant women with severe anemia as compared to those with moderate anemia. We, therefore, concluded that IVIS therapy may be introduced in the routine ANC clinic of a secondary care health facility.
